# Blockage of angiotensin II type I receptor decreases the synthesis of growth factors and induces apoptosis in C6 cultured cells and C6 rat glioma

**DOI:** 10.1038/sj.bjc.6602483

**Published:** 2005-03-22

**Authors:** O Arrieta, P Guevara, E Escobar, R García-Navarrete, B Pineda, J Sotelo

**Affiliations:** 1 Neuroimmunology Unit of the National Institute of Neurology and Neurosurgery of Mexico, Insurgentes Sur 3877, 14269 Mexico City, Mexico

**Keywords:** angiotensin, AT_1_, glioblastoma, apoptosis, growth factors

## Abstract

Angiotensin II (Ang II) is a main effector peptide in the renin–angiotensin system and participates in the regulation of vascular tone. It also has a role in the expression of growth factors that induce neovascularisation which is closely associated to the growth of malignant gliomas. We have shown that the selective blockage of the AT_1_ receptor of angiotensin inhibites tumour growth, cell proliferation and angiogenesis of C6 rat glioma. The aim of this study was to study the effects of the blockage of AT_1_ receptor on the synthesis of growth factors, and in the genesis of apoptosis in cultured C6 glioma cells and in rats with C6 glioma. Administration of losartan at doses of 40 or 80 mg kg^−1^ to rats with C6 glioma significantly decreased tumoral volume and production of platelet-derived growth factor, vascular endothelial growth factor and basic fibroblast growth factor. It also induced apoptosis in a dose-dependent manner. Administration of Ang II increased cell proliferation of cultured C6 cells which decreased by the administration of losartan. Our results suggest that the selective blockage of AT_1_ diminishes tumoral growth through inhibition of growth factors and promotion of apoptosis.

The renin angiotensin aldosterone system (RAAS) has an important role in the regulation of blood pressure and fluid electrolyte balance. Angiotensin II (Ang II), a major participant in the RAAS, was initially described as a vasoconstrictor, but recent studies have revealed that it also participates in cell growth, cell differentiation and apoptosis (Stoll *et al*, 1995; Escobar *et al*, 2004), and has a role in cell migration and conformation of the extracellular matrix (Coker *et al*, 2001). Some reports indicate that Ang II induce neovascularisation (Fernandez *et al*, 1985; Le Noble *et al*, 1991; Andrade *et al*, 1996) due to stimulation of growth factors, such as platelet-derived growth factor (PDGF) (Khachigian *et al*, 2000; Cook *et al*, 2002), transforming growth factor beta *β* (TGF*β*) (Kagami *et al*, 1994; Ohta *et al*, 1994; Hamaguchi *et al*, 1999; Weigert *et al*, 2002), insulin-like growth factor 1 (IGF-1) (Brink *et al*, 1999; Haddad *et al*, 2003), basic fibroblast growth factor (bFGF) (Peng *et al*, 2001), vascular endothelial growth factor (VEGF) (Otani *et al*, 1998; Tamarat *et al*, 2002) and angiopoietin 2 (Otani *et al*, 2001). Angiotensin II also induces the expression of proto-oncogenes in smooth vascular muscle cells, including *c-fos*, *c-jun*, *c-myc*, *erg-1*, *VL-30*, and the activator of the protein 1 complex (Cook *et al*, 2002), Interestingly, many of these effects are inhibited by the blockage of the AT_1_ receptor (Fujiyama *et al*, 2001). Angiotensin II has a dual and paradoxical intervention in apoptosis through its receptors AT_1_ functioning as antiapoptotic and AT_2_ functioning as proapoptotic (Stoll *et al*, 1995).

Experimentally, the RAAS has been associated to proliferation of tumours; Ang II receptors have been found on the cell surface and cytoplasm of human tumours such as breast cancer (Guerra *et al*, 1993; Inwang *et al*, 1997; Berry *et al*, 2000; Muscella *et al*, 2002), hepatic carcinoma (Yoshiji *et al*, 2002), renal carcinoma (Hii *et al*, 1998; Miyajima *et al*, 2002), melanoma (Egami *et al*, 2003), colorectal carcinoma, squamous cell carcinoma (Takeda and Kondo, 2001), pancreas cancer (Fujimoto *et al*, 2001) and sarcomas (Volpert *et al*, 1996; Fujita *et al*, 2002).

Glioma is the most frequent primary tumour of the brain. Malignant gliomas are still associated with poor prognosis, the mean survival time of patients with glioblastoma multiforme (GBM) is 1 year, and it has not changed significantly for the last three decades (Lopez-Gonzalez and Sotelo, 2000). Glioblastoma multiforme is accompanied by extensive angiogenesis which is essential for tumoral growth and invasiveness; it also produces a vast amount of growth factors such as PDGF, VEGF, HGF and FGF (Strugar *et al*, 1995; Arrieta *et al*, 1998, 2002; Moriyama *et al*, 1999; Schmidt *et al*, 1999). In brain parenchyma there is a local RAAS, some neurons, glial cells and glioma cells express renin, angiotensinogen and receptors for Ang II (AT_1_ and AT_2_) (Ganong, 1984, Ariza *et al*, 1988; Fogarty *et al*, 2002; Juillerat-Jeanneret *et al*, 2004). We have previously shown that the blockage of AT_1_ receptors in rats with C6 glioma inhibited tumour growth, cell proliferation and angiogenesis (Rivera *et al*, 2001). The aim of this study was to determine *in vitro* (in cultured C6 glioma cells) and *in vivo* (in rats with C6 glioma) the effects of the blockage of the AT_1_ receptor on the synthesis of growth factors and its relation with cell proliferation and apoptosis.

## MATERIALS AND METHODS

### Glioma induction

C6 glioma cells (American Tissue Culture Collection Rockville, MD, USA) were cultured under sterile conditions at 37°C in a humid environment with 5% CO_2_ in Dulbecco's modified Eagle's medium (DME) (Sigma chemical company, Saint Louis Missouri, USA) supplemented with 10% foetal bovine serum (GIBCO, New York, USA). After the cultures became confluent, the cells were washed with saline solution and harvested; 10^7^ C6 cells from this source were inoculated intraperitoneally in a male Wistar rat; 2 weeks later, a large tumour was obtained (Guevara and Sotelo, 1999), it was mechanically dispersed in saline solution (1 : 1) at 4°C, 10^7^ cells from this source were subcutaneously injected in the left thigh of 40-day-old Wistar rats (Arrieta *et al*, 2001). All animals developed a noticeable tumour within 2 weeks. All animals used in this study were handled in accordance to the guidelines of the coordinating committee on cancer research for the welfare of animals with experimental neoplasms.

### Blockage of AT_1_*in vivo*

When the tumour had reached a diameter of 1.5 cm, the rats were randomly assigned either to the control group (*n*=40), to the losartan 40 mg Kg^−1^ group (L40) (*n*=40), or to the losartan 80  mg Kg^−1^ group (L80) (*n*=40). Losartan (Merck-Sharp & Dohme, Mexico) was given orally, once a day, for 30 days. At the end of the experiment, 20 rats from each group were anaesthetised and perfused by intracardiac route with 10% formalin in saline solution for histological study. Before perfusion, the animals were bled by intracardiac puncture to analyse haematological and chemical parameters in blood; the body weight of animals from all groups remained similar throughout the study. The tumour was dissected and its volume was determined by water displacement. The tumours from nonperfused animals were kept at −70°C until analysis.

### Histological analysis and apoptosis *in vivo*

For microscopic study, the tumour was embedded in paraffin: 5 *μ*m sections were stained with haematoxylin and eosin. Apoptosis was detected by DNA fragmentation using TdT incorporation of nucleotides on 3′ ends of DNA (TUNEL technique). Briefly, 5 *μ*m of glioma C6 tumour sections was dewaxed and immersed in 3% H_2_O_2_ to block endogenous peroxidase; after washing with distilled water, proteinase K (20 *μ*g ml^−1^) was applied to the specimens for 15 min at room temperature. Detection of apoptosis *in situ* was made by ApopTag Peroxidase (Oncor, Gaithersburg, MD, USA) that detects fragmented DNA and was performed according to the manufactureŕs conditions. Sections were counterstained with methyl-green. Positive control sections were prepared by nicking DNA with DNase and negative control sections were prepared by substituting with distilled water for working strength TdT enzyme. The proportion of apoptotic cells was expressed as apoptotic rate, which represented the number of apoptotic cells among 1000 glioma C6 nucleated cells, excluding segmented neutrophils due to their short lifespan.

### Determination of growth factors *in vivo*

In all, 10 samples from each experimental group were unfrozen, weighted, and homogenised at 4°C (1 : 1) in saline solution; 70 *μ*g ml^−1^ of phenylmethylsulphonyl fluoride (PMSF) was added for inhibition of proteases, the lysate was centrifuged at 6500 g for 20 min and kept at −70°C until analysis. Tissue contents of bFGF, VEGF, PDGF and HGF were measured by enzyme-linked immunosorbent assay (ELISA) (R&D system, Minneapolis, MN, USA). Each sample was assayed by duplicate and reported as means±s.d.

### Treatment of C6 cells in culture

We separated C6 glioma cells in four groups: control cells (without treatment); cultures treated with Ang II (10^−7^ M); cultures treated with Losartan (10^−5^ M) (Stroth *et al*, 2000) and cultures treated with both Ang II and Losartan (L–A) at the same doses. All cultures were kept in a serum-free medium at 37°C, in humidified environment with 5% CO_2_. The culture medium used for cells incubated for long periods was changed every 24 h containing either Ang II, losartan or Ang ll plus losartan at the mentioned doses.

### Cell viability of C6 glioma in culture

For quantification of cell viability, 6.5 × 10^5^ glioma cells were cultured in microtitre plates (96 wells) with 100 *μ*l culture medium and incubated for 24 h in a humidified atmosphere; 10 *μ*l of tetrazolium salts (MTT) was added to each well and left for 4 h; these salts were cleaved into a coloured formazan product by metabolically active cells; afterwards 100 *μ*l of solubilisation solution was added, the plates were allowed to stand overnight inside the incubator. After checking for complete solubilisation of the purple formazan crystals, the absorbance was measured at 570 nm, the reference wavelength was 650 nm. We obtained the percentage of viable cells from three assays, the control was used as reference (100%). Two replicates were perfomed using the same dosages.

### Apoptotic rate *in vitro* by flow cytometry

For quantification of apoptosis by flow cytometry, 10^6^ C^6^ glioma cells were trypsinised and transferred to 1.5 ml Eppendorf tubes and cultured with DME culture medium either alone (controls) or with Losartan 10^−5^ M, or with Ang II 10^−7^ M, or with the mixture of losartan/Ang II (LA). Cultured cells were maintained at 37°C with 5% CO_2_ during 6, 12, 24, 48 or 96 h. The culture medium of those experimental groups which needed a long time of incubation was changed every day. At the end of the incubation period, the cells were centrifuged at 2000 **g**, the supernatant was discarded. Induced apoptosis was detected by flow-cytometric analysis of the permeabilised, propidium-iodide-stained cells (Telford *et al*, 1992). Samples (10^6^ cells ml^−1^) were washed once in PBS and the pellets were re-suspended in 80% ethanol at 4°C for 60 min. To detect apoptosis by flow cytometry, fixed cells were centrifuged, re-suspended in 1 ml PBS, and kept at 37°C for 20 min before staining with a solution of 0.1% Triton X-100, 0.1 mM EDTA(Na)_2_, 5 U ml^−1^ RNase and 20 mg ml^−1^ propidium iodide in PBS. Samples were stored in the dark at room temperature and analysed with a Facscalibur Registered Trademark (Becton Dickinson & Co., San Jose, CA, USA). Using the cell quest software (San Jose CA, USA), cell number (10^4^) was detected. Cell percentages in the different phases of the cell cycle were estimated according to the Fox's method (Lacombe *et al*, 1988). The assays were made by triplicate.

### Apoptotic rate *in vitro* by ELISA

For quantification of apoptosis by ELISA, 10^5^ C^6^ glioma cells were trypsinised and transferred to 1.5 ml Eppendorf tubes and cultured with DME culture medium either alone (controls) or with Losartan 10^−5^ M, or with Ang II 10^−7^ M, or with the mixture of LA. Cultured cells were maintained at 37°C with 5% CO_2_ during 6, 12, 24, 48 or 96 h. The culture medium of those experimental groups which needed a long time of incubation was changed every day. At the end of the incubation period, the cells were centrifuged at 2000**g**, the supernatant was discarded and the cells were re-suspended in 1 ml of culture medium with 10% DMSO and kept in liquid nitrogen until processed. At that time, the vials were warmed at 37°C and the cells were washed with culture medium by centrifugation at 2000**g**. The cells were then re-suspended with incubation buffer (Cell Death detection ELISA Kit, Boehringer Mannheim catalogue: 1544675) at 4°C during 30 min. The cellular lysate was centrifuged at 20 000**g** for 10 min and 400 *μ*l of the supernatant were removed (cytoplasmic fraction) and diluted 1 : 10 with incubation buffer, absorbance was measured at 405 nm, the substrate solution was used as blank. The assays were made by triplicate; 10^6^ C6 cells in hypertonic buffer and 10^6^ C6 cells with camptotensin were used as positive controls.

### Statistical analysis

Values were expressed as means±s.d.; for the quantification of apoptotic rate, the kappa value was used as the interobserver variability value. Statistical analysis was made using SPSS v10 software. Comparisons between groups were made by ANOVA and Tukey tests. Statistical significance was set at a *P*-value of 0.05.

## RESULTS

### Effect of the blockage of AT_1_ receptor on tumoral growth and apoptotic rate *in vivo*

**Figure 1:**
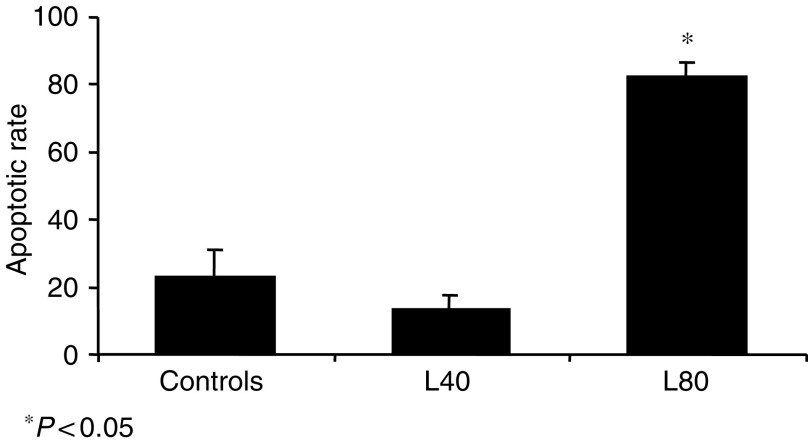
Effect of losartan administration at doses of 40 and 80 mg Kg^−1^ on the apoptotic rate in tissue sections of C6 glioma (TUNEL stain). A significant increase of the apoptosis was observed with losartan treatment at 80 mg Kg^−1^.

All animals survived until the end of the experiment. In all controls, the tumour grew to a very large size (over 6 cm diameter); there was no case of spontaneous involution. When compared to controls (mean volume: 55±10 cm^3^) a significant decrease in tumour volume was seen in animals treated with L40 (30±7 cm^3^) and with L80 (19±9 cm^3^) (*P*<0.05 and 0.01, respectively). Results were similar to those from our previous report (Rivera *et al*, 2001). The apoptotic rate was higher in the L80 group (84.2±4.1) than in controls (23±8) or in the L40 group (13.6±4.3) (*P*<0.01); no differences were seen when L40 group was compared with controls (Figure 1). Comparisons of haematological and chemical blood parameters measured at the end of the study showed no differences between groups.

### Growth factors in neoplastic tissue

**Figure 2:**
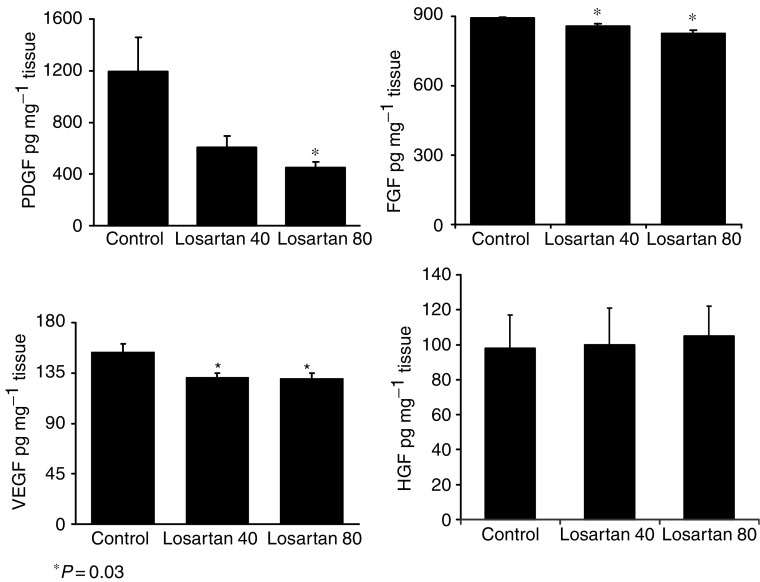
Effect of the blockage of AT_1_ receptor with losartan on the contents of PDGF, FGF, VEGF and HGF in C6 rat glioma. A significant reduction in the concentration of PDGF, FGF and VEGF was obtained with losartan treatment.

Mean contents of PDGF, bFGF and VEGF were higher in tumours from control animals (PDGF=1,195±261, bFGF=893±4, and VEGF=154±7 pg mg^−1^ of tissue) than in tumours of animals treated with L40 (PDGF=609±84, *P*=0.05; bFGF=857±12, *P*=0.035; and VEGF=131±4 pg mg^−1^, *P*=0.011) and those treated with L80 (PDGF=451±45, *P*=0.018; bFGF=827±14, *P*=0.001; and VEGF=105±19 pg mg^−1^, *P*=0.013). However, no significant differences were seen on HGF contents between controls (97±20 pg mg^−1^) and L40-(100±29 pg mg^−1^) or L80- treated rats (105±19 pg mg^−1^) (*P*=0.65) (Figure 2).

### Effects of Ang II, losartan and LA on cell cultures

**Figure 3:**
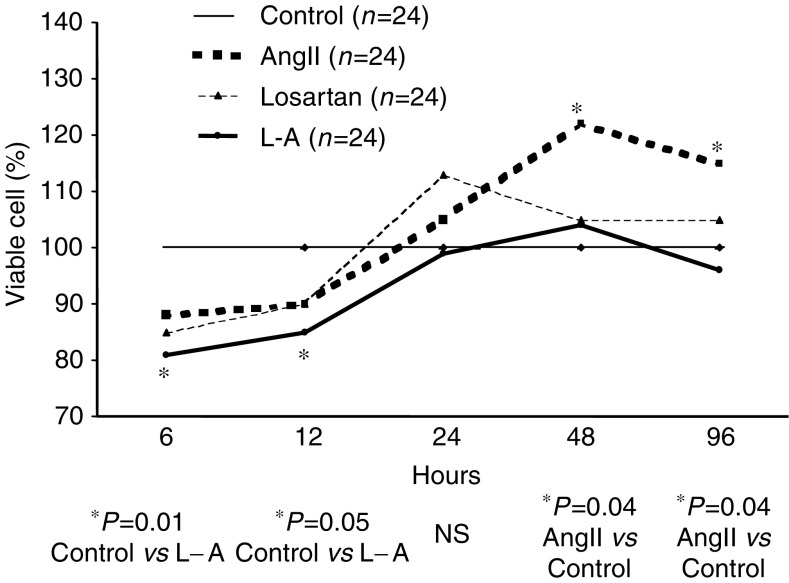
Effects of the administration of Ang II (10^−7^ M), losartan (10^−5^ M), or Ang II plus losartan on cultured C6 cells. During the initial 12 h of treatment, there was a significant reduction of viability in the cells treated with Ang II plus losartan; in contrast, after 48 h, a significant increase of viability was observed in the cells treated with Ang II alone.

The percentage of viable cells was significantly smaller in cultures treated with L–A at 6 and 12 h as compared with controls (*P*=0.01 and 0.05, respectively); however, no differences were seen later, at 24, 48 and 96 h. The percentage of viable cells was higher in cells cultured with Ang II at 48 and 96 h as compared to controls (*P*=0.04 for both determinations). Treatment with Losartan had no effect on cell viability (Figure 3).

### Apoptosis rate *in vitro* by flow cytometry

**Figure 4:**
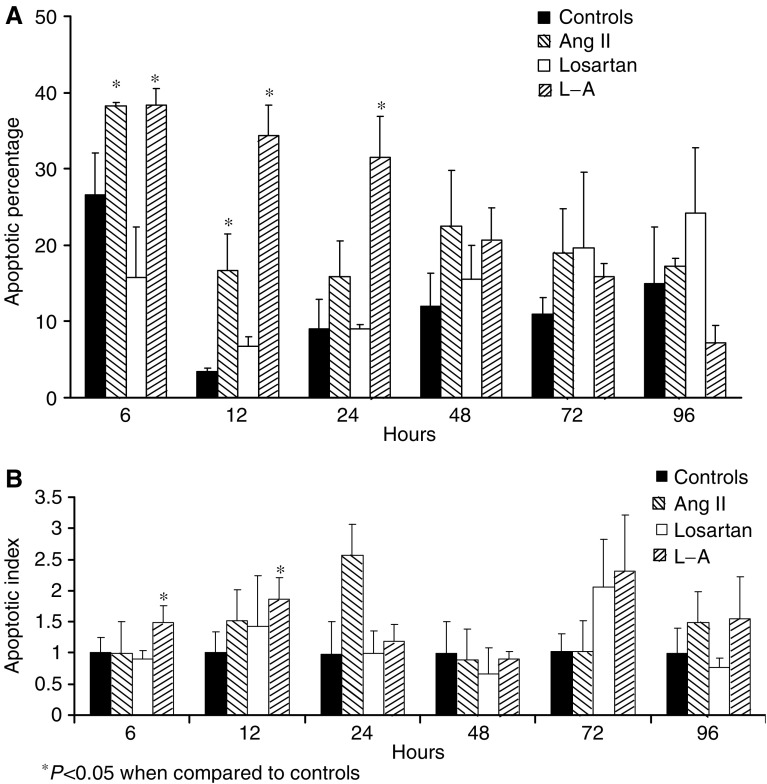
Effects of Ang II (10^−7^ M), losartan (10^−5^ M) and Ang II plus losartan on apoptosis in cultured glioma C6 cells measured either by flow cytometry (**A**) or by ELISA (**B**). In both determinations the cells treated with losartan plus Ang II showed an increase of apoptosis during the initial hours after treatment.

A significative increase of apoptosis was observed in cells treated with L–A particularly at 6 h as compared to controls (*P*=0.05) and with the losartan group (*P*=0.012), and at 12 h as compared with the groups Ang II (*P*=0.007) and losartan (*P*=0.001), which was maintained at 24 h with losartan (*P*=0.007). No differences were found at 48, 72 and 96 h (Figure 4A).

### Apoptosis rate *in vitro* by ELISA

Cells cultured with Ang II showed increased apoptotic rate at 6 h when compared with losartan (*P*=0.012) and at 12 h when compared to controls (*P*=0.012). When compared with controls, apoptosis was increased in cells treated with L–A (*P*=0.007), with losartan (*P*=0.003) and with Ang II (*P*=0.011) at 6 h, as well as the cells treated with L–A at 12 h (*P*=0.05). No differences were found at 24, 48, 72 and 96 h (Figure 4B).

## DISCUSSION

Malignant gliomas contain large quantities of VEGF, PDGF, bFGF and HGF, as well as their receptors (Stefanik *et al*, 1991; Westermark *et al*, 1995; Arrieta *et al*, 2002; Steiner *et al*, 2003); their concentrations are related to vascular density, radioresistance, cell proliferation, degree of malignancy and patient́s survival (Westermark *et al*, 1995; Gorski *et al*, 1999). Angiotensin II stimulates the production of these growth factors (Peng *et al*, 2001; Tamarat *et al*, 2002); this effect can be blocked by AT_1_ antagonists but not by AT_2_ antagonists (Tamarat *et al*, 2002). Normal and neoplasic astrocytes including C6 glioma cells express AT_1_ and AT_2_ receptors for Ang II (Rivera *et al*, 2001; Fogarty *et al*, 2002). We have previously shown in experimentally induced C6 glioma in rats that the selective blockage of AT_1_ receptor reduces cell proliferation, angiogenesis and tumour growth (Rivera *et al*, 2001). However, the participative mechanisms were unclear. In this study, we found that the blockage of AT_1_ decreases the synthesis of the growth factors VEGF, PDGF and bFGF, coincident with the reduction of tumour size, cell proliferation and vascular density. The effect of losartan in the synthesis of growth factors is more intense in PDGF, which is inhibited with the blockage of AT1 in several experimental models; nonetheless, VEGF and FGF syntheses are also statistically reduced. However, the reduction of angiogenesis previously reported might be a consequence of VEGF and FGF inhibition due to a blockage of PDGF’s stimuli, this would explain why the effect is not dose-dependent on losartan. Although HGF is also overexpressed in malignant gliomas and related to the degree of malignancy (Arrieta *et al*, 2002) and Ang II also participates in its synthesis (Matsumoto *et al*, 2003), in this study no effect on HGF contents was observed after blockage of the AT_1_ receptor.

Additionally, our results *in vitro* show that the reduction on cell viability within the first h of Ang II plus losartan administration was also associated with the induction of apoptosis but only during the first 12 h, whereas the administration of Ang II alone stimulates cell proliferation during 24 h after exposure. Increase of apoptosis in glioma C6 *in vivo* was seen only at high doses of losartan (80 mg Kg^−1^); this effect was also obtained in cultured C6 cells with the simultaneous administration of losartan and Ang II; it seems that the selective blockage of AT_1_ may lead to disequilibrium of AT_1_/AT_2_ relation that promotes AT_2_ receptor stimulation which, in turn, could increase its proapoptotic effects. It is also likely that, in this condition, the presence of Ang II promotes apoptosis, as this effect is prevented by deletion of the AT_2_ receptor gene (Yamada *et al*, 1996). Our findings are in agreement with studies in PC12W cells and neurons from newborn rats, showing that apoptosis is increased either by the selective stimulation of AT_2_ receptor or by the blockage of AT_1_ receptor simultaneous to the administration of Ang ll (Stoll *et al*, 1995; Yamada *et al*, 1996; Goldenberg *et al*, 2001; Suzuki *et al*, 2002).

Subucutaneous C6 glioma, in contrast to brain glioma, allowed us to measure tumour growth for a longer time and to determine a potential therapeutic effect when the drug is administered for long periods. However, similar results are to be expected in brain glioma as the blood-brain barrier is interrupted in brain tumours, facilitating the entrance of drugs into the tumour.

Angiogenesis and apoptosis are primordial features of malignant tumours, constituting attractive therapeutic targets; drugs that inhibit angiogenesis or promote apoptosis could be associated to cytotoxic agents to improve antitumoral therapy. The fact that multifunctional hormonal systems, such as the renin–angiotensin–aldosterone system, influence tumour growth and angiogenesis provides interesting pathways to the study of cancer. The antineoplastic activity obtained by the selective blockage of AT_1_ in malignant glioma seems to be mediated by two different mechanisms, inhibition of the synthesis of growth factors and promotion of apoptosis, providing a potential therapeutic adjuvant for malignant gliomas.

## References

[bib1] Andrade SP, Cardoso CC, Machado RD, Beraldo WT (1996) Angiotensin-II induced angiogenesis in sponge implants in mice. Int J Microcirc Clin Exp 16: 302–307 9049708 10.1159/000179189

[bib2] Ariza A, Fernandez LA, Inagami T, Kim JH, Manuelidis EE (1988) Renin in glioblastoma multiforme and its role in neovascularization. Am J Clin Pathol 90: 341–437 10.1093/ajcp/90.4.437 2459955

[bib3] Arrieta O, Garcia E, Guevara P, Garcia NR, Ondarza R, Rembao D, Sotelo J (2002) Hepatocyte growth factor is associated with poor prognosis of malignant gliomas and is a predictor for recurrence of meningioma. Cancer 94: 3210–3218 12115353 10.1002/cncr.10594

[bib4] Arrieta O, Guevara P, Reyes S, Ortiz A, Rembao D, Sotelo J (1998) Protamine inhibits angiogenesis and growth of C6 rat glioma; a synergistic effect when combined with carmustine. Eur J Cancer 34: 2102–2106 10.1016/s0959-8049(98)00244-5 10070318

[bib5] Arrieta O, Guevara P, Reyes S, Palencia G, Rivera E, Sotelo J (2001) Paradoxical effect of aspirin on the growth of C6 rat glioma and on time of development of ENU-induced tumors of the nervous system. J Cancer Res Clin Oncol 127: 681–686 11710598 10.1007/s004320100267 PMC12164671

[bib6] Berry MG, Goode AW, Puddefoot JR, Vinson GP, Carpenter R (2000) Integrin *β*1 upregulation in MCF-7 breast cancer cells by angiotensin II. Eur J Surg Oncol 26: 25–29 10718175 10.1053/ejso.1999.0735

[bib7] Brink M, Chrast J, Price SR, Mitch WE, Delafontaine P (1999) Angiotensin II stimulates gene expression of cardiac Insulin-like growth factor I and its receptor through effects on blood pressure and food intake. Hypertension 34: 1053–1059 10567181 10.1161/01.hyp.34.5.1053

[bib8] Coker ML, Jolly JR, Joffs C, Etoh T, Holder JR, Bond BR, Spinale FG (2001) Matrix metalloproteinase expression and activity in isolated myocytes after neurohormonal stimulation. Am J Physiol Heart Circ Physiol 281: H543–H551 11454555 10.1152/ajpheart.2001.281.2.H543

[bib9] Cook JL, Giardina JF, Zhang Z, Re RN (2002) Intracellular ANG II increases the long isoform of PDGF mRNA in rat hepatoma cells. J Mol Cell Cardiol 34: 1525–1537 12431451 10.1006/jmcc.2002.2106

[bib10] Egami K, Murohara T, Shimada T, Sasaki K, Shintani S, Sugaya T, Ishii M, Akagi T, Ikeda H, Matsuishi T, Imaizumi T (2003) Role of host angiotensin II type 1 receptor in tumor angiogenesis and growth. J Clin Invest 112: 67–75 12840060 10.1172/JCI16645 PMC162282

[bib11] Escobar E, Rodríguez-Reyna T, Arrieta O, Sotelo J (2004) Angiotensin II, cell proliferation and angiogenesis regulator: Biologic and therapeutic implications in cancer. Curr Vasc Pharmacol 2: 385–399 15320819 10.2174/1570161043385556

[bib12] Fernandez LA, Twickler J, Mead A (1985) Neovascularization produced by angiotensin II. J Lab Clin Med 105: 141–145 2579174

[bib13] Fogarty DJ, Sánchez GV, Matute C (2002) Multiple angiotensin receptor subtypes in normal and tumor astrocytes *in vitro*. Glia 39: 304–313 12203396 10.1002/glia.10117

[bib14] Fujimoto Y, Sasaki T, Tsuchida A, Chayama K (2001) ANG II type 1 receptor expression in human pancreatic cancer and growth inhibition by ANG II type 1 receptor antagonist. FEBS Lett 495: 197–200 11334891 10.1016/s0014-5793(01)02377-8

[bib15] Fujita M, Hayashi I, Yamashina S, Itoman M, Majima M (2002) Blockade of angiotensin AT1a receptor signaling reduces tumor growth, angiogenesis, and metastasis. Biochem Biophys Res Commun 294: 441–447 12051731 10.1016/S0006-291X(02)00496-5

[bib16] Fujiyama S, Matsubara H, Nozawa Y, Maruyama K, Mori Y, Tsutsumi Y, Masaki H, Uchiyama Y, Koyama Y, Nose A, Iba O, Tateishi E, Ogata N, Jyo N, Higashiyama S, Iwasaka T (2001) Angiotensin AT(1) and AT(2) receptors differentially regulate angiopoietin-2 and vascular endothelial growth factor expression and angiogenesis by modulating heparin binding-epidermal growth factor (EGF)-mediated EGF receptor transactivation. Circ Res 88: 22–29 11139469 10.1161/01.res.88.1.22

[bib17] Ganong WF (1984) The brain renin–angiotensin system. Ann Rev Physiol 46: 17–31 6324655 10.1146/annurev.ph.46.030184.000313

[bib18] Goldenberg I, Grossman E, Jacobson KA, Shneyvays V, Shainberg A (2001) Angiotensin II-induced apoptosis in rat cardiomyocyte culture: a possible role of AT1 and AT2 receptors. J Hypertens 19: 1681–1689 11564990 10.1097/00004872-200109000-00022 PMC7458782

[bib19] Gorski DH, Beckett MA, Jaskowiak NT, Calvin DP, Mauceri HJ, Salloum RM, Seetharam S, Koons A, Hari DM, Kufe DW, Weichselbaum RR (1999) Blockage of the vascular endothelial growth factor stress response increases the antitumor effects of ionizing radiation. Cancer Res 59: 3374–3378 10416597

[bib20] Guerra FK, Ciuffo GM, Elizalde PV, Charreau EH, Saavedra JM (1993) Enhanced expression of angiotensin II receptor subtypes and angiotensin converting enzyme in medroxy progesterone-induced mouse mammary adenocarcinomas. Biochem Biophys Res Commun 193: 93–99 8389152 10.1006/bbrc.1993.1594

[bib21] Guevara P, Sotelo J (1999) C6 rat glioma grown into the peritoneal cavity, a large source of tumoral cells for subcutaneous transplant of glioma. J Neurooncol 44: 91–92 10582675 10.1023/a:1006112422132

[bib22] Haddad GE, Blackwell K, Bikhazi A (2003) Regulation of insulin-like growth factor-1 by the renin–angiotensin system during regression of cardiac eccentric hypertrophy through angiotensin-converting enzyme inhibitor and AT1 antagonist. Can J Physiol Pharmacol 81: 142–149 12710528 10.1139/y02-154

[bib23] Hamaguchi A, Kim S, Izumi Y, Zhan Y, Yamanaka S, Iwao H (1999) Contribution of extracellular signal-regulated kinase to angiotensin II-induced transforming growth factor-beta 1 expression in vascular smooth muscle cells. Hypertension 34: 126–131 10406835 10.1161/01.hyp.34.1.126

[bib24] Hii SI, Nicol DL, Gotley DC, Thompson LC, Green MK, Jonsson JR (1998) Captopril inhibits tumour growth in a xenograft model of human renal cell carcinoma. Br J Cancer 77: 880–883 9528828 10.1038/bjc.1998.145 PMC2150111

[bib25] Inwang ER, Puddefoot JR, Brown CL, Goode AW, Marsigliante S, Ho MM, Payne JG, Vinson GP (1997) Angiotensin II type 1 receptor expression in human breast tissues. Br J Cancer 75: 1279–1283 9155046 10.1038/bjc.1997.217 PMC2228240

[bib26] Juillerat-Jeanneret L, Celerier J, Chapuis Bernasconi C, Nguyen G, Wostl W, Maerki HP, Janzer RC, Corvol P, Gasc JM (2004) Renin and angiotensinogen expression and functions in growth and apoptosis of human glioblastoma. Br J Cancer 90: 1059–1068 14997208 10.1038/sj.bjc.6601646 PMC2409624

[bib27] Kagami S, Border WA, Miller DE, Noble NA (1994) Angiotensin II stimulates extracellular matrix protein synthesis through induction of transforming growth factor-beta expression in rat glomerular mesangial cells. J Clin Invest 93: 2431–2437 8200978 10.1172/JCI117251 PMC294451

[bib28] Khachigian LM, Takuwa Y, Collins T (2000) Mechanisms of angiotensin II-induced platelet-derived growth factor gene expression. Mol Cell Biochem 212: 183–186 11108150

[bib29] Lacombe F, Belloc F, Bernard P, Boisseau MR (1988) Evaluation of four methods of DNA distribution data analysis based on bromodeoxyuridine/DNA bivariate data. Cytometry 9: 245–253 3378459 10.1002/cyto.990090310

[bib30] Le Noble FA, Hekking JW, Van Straaten HW, Slaaf DW, Struyker-Boudier HA (1991) Angiotensin II stimulates angiogenesis in the chorioallantoic membrane of the chick embryo. Eur J Pharmacol 195: 305–306 1874278 10.1016/0014-2999(91)90552-2

[bib31] Lopez-Gonzalez MA, Sotelo J (2000) Brain tumors in mexico: characteristics and prognosis of glioblastoma. Surg Neurol 53: 157–162 10713194 10.1016/s0090-3019(99)00177-9

[bib32] Matsumoto K, Morishita R, Tomita N, Moriguchi A, Komai N, Aoki M, Matsumoto K, Nakamura T, Higaki J, Ogihara T (2003) Improvement of endothelial dysfunction by angiotensin II blockade accompanied by induction of vascular hepatocyte growth factor system in diabetic spontaneously hypertensive rats. Heart Vessels 18: 18–25 12644877 10.1007/s003800300003

[bib33] Miyajima A, Kosaka T, Asano T, Seta K, Kawai T, Hayakawa M (2002) ANG II type I antagonist prevents pulmonary metastasis of murine renal cancer by inhibiting tumor angiogenesis. Cancer Res 62: 4176–4179 12154013

[bib34] Moriyama T, Kataoka H, Koono M, Wakaisaka S (1999) Expression of hepatocyte growth factor/scatter factor and its receptor c-MET in brain tumors: evidence for a role in progression of astrocytic tumors. Int J Mol Med 3: 531–536 10202187 10.3892/ijmm.3.5.531

[bib35] Muscella A, Greco S, Elia MG, Storelli C, Marsigliante S (2002) Angiotensin II stimulation of Na+/K+ATPase activity and cell growth by calcium-independent pathway in MCF-7 breast cancer cells. J Endocrinol 173: 315–323 12010639 10.1677/joe.0.1730315

[bib36] Ohta K, Kim S, Hamaguchi A, Yukimara T, Miura K, Takaori K, Iwao H (1994) Role of angiotensin II in extracelluar matrix and transforming growth factor-beta 1 expression in hypertensive rats. Eur J Pharmacol 269: 115–119 7828653 10.1016/0922-4106(94)90033-7

[bib37] Otani A, Takagi H, Oh H, Koyama S, Honda Y (2001) Angiotensin II induces expression of the Tie2 receptor ligand, angiopoietin 2, in bovine retinal endothelial cells. Diabetes 50: 867–875 11289054 10.2337/diabetes.50.4.867

[bib38] Otani A, Takagi H, Suzuma K, Honda Y (1998) Angiotensin II potentiates vascular endothelial growth factor-induced angiogenic activity in retinal microcapillary endothelial cells. Circ Res 82: 619–628 9529167 10.1161/01.res.82.5.619

[bib39] Peng H, Moffett J, Myers J, Fang X, Stachowiak EK, Maher P, Kratz E, Hines J, Fluharty SJ, Mizukoshi E, Bloom DC, Stachowiak MK (2001) Novel nuclear signaling pathway mediates activation of fibroblast growth factor-2 gene by type 1 and type 2 angiotensin II receptors. Mol Biol Cell 12: 449–462 11179427 10.1091/mbc.12.2.449 PMC30955

[bib40] Rivera E, Arrieta O, Guevara P, Duarte-Rojo A, Sotelo J (2001) AT1 receptor is present in glioma cells; its blockage reduces the growth of rat glioma. Br J Cancer 85: 1396–1399 11720480 10.1054/bjoc.2001.2102 PMC2375243

[bib41] Schmidt NO, Westphal M, Hagel C, Ergun S, Stavrou D, Rosen EM, Lamszus K (1999) Levels of vascular endothelial growth factor, hepatocyte growth factor/scatter factor and basic fibroblast growth factor in human gliomas and their relation to angiogenesis. Int J Cancer 84: 10–18 9988225 10.1002/(sici)1097-0215(19990219)84:1<10::aid-ijc3>3.0.co;2-l

[bib42] Stefanik DF, Rizkalla LR, Soi A, Goldblatt SA, Rizkalla WM (1991) Acidic and basic fibroblast growth factors are present in glioblastoma multiforme. Cancer Res 51: 5760–5765 1717153

[bib43] Steiner HH, Karcher S, Mueller MM, Nalbantis E, Kunze S, Herold-Mende C (2003) Autocrine pathways of the vascular endothelial growth factor (VEGF) in glioblastoma multiforme: clinical relevance of radiation-induced increase of VEGF levels. J Neurooncol 66: 129–138 10.1023/b:neon.0000013495.08168.8f 15015778

[bib44] Stoll M, Steckelings UM, Paul M, Bottarri SP, Metzger R, Unger T (1995) The angiotensin AT2-receptor mediates inhibition of cell proliferation in coronary endothelial cells. J Clin Invest 95: 651–657 7860748 10.1172/JCI117710 PMC295531

[bib45] Stroth U, Blume A, Mielke K, Unger T (2000) Angiotensin AT(2) receptor stimulates ERK1 and ERK2 in quiescent but inhibits ERK in NGF-stimulated PC12 cells. Brain Res Mol Brain Res 78: 175–180 10891597 10.1016/s0169-328x(00)00093-0

[bib46] Strugar JG, Criscuolo GR, Rothbart D, Harrington WN (1995) Vascular endothelial growth/permeability factor expression in human glioma specimens: correlation with vasogenic brain edema and tumor-associated cysts. J Neurosurg 83: 682–689 7674019 10.3171/jns.1995.83.4.0682

[bib47] Suzuki J, Iwai M, Nakagami H, Wu L, Chen R, Sugaya T, Hamada M, Hiwada K, Horiuchi M (2002) Role of angiotensin II – regulates apoptosis through distinct AT1 and AT2 receptors in neointimal formation. Circulation 106: 847–853 12176959 10.1161/01.cir.0000024103.04821.86

[bib48] Takeda H, Kondo S (2001) Differences between squamous cell carcinoma and keratoacanthoma in angiotensin type-1 receptor expression. Am J Pathol 158: 1633–1637 11337361 10.1016/S0002-9440(10)64119-3 PMC1891940

[bib49] Tamarat R, Silvestre JS, Durie M, Levy BI (2002) Angiotensin II angiogenic efect *in vivo* involves vascular endothelial growth factor- and inflammation-related pathways. Lab Invest 82: 747–756 12065685 10.1097/01.lab.0000017372.76297.eb

[bib50] Telford WG, King LE, Fraker PJ (1992) Comparative evaluation of several DNA binding dyes in the detection of apoptosis-associated chromatin degradation by flow cytometry. Cytometry 13: 137–143 1372208 10.1002/cyto.990130205

[bib51] Volpert OV, Ward WF, Lingen MW, Chesler L, Solt DB, Johnson MD, Molteni A, Polverini PJ, Bouck NP (1996) Captopril inhibits angiogenesis and slows the growth of experimental tumors in rats. J Clin Invest 98: 671–679 8698858 10.1172/JCI118838 PMC507476

[bib52] Weigert C, Brodbeck K, Klopfer K, Haring U, Schleicher ED (2002) Angiotensin II induces human TGF-beta 1 promoter activation: similarity to hyperglycaemia. Diabetologia 45: 890–898 12107734 10.1007/s00125-002-0843-4

[bib53] Westermark B, Heldin CH, Nister M (1995) Platelet-derived growth factor in human glioma. Glia 15: 257–563 8586462 10.1002/glia.440150307

[bib54] Yamada T, Horiuchi M, Dzau VJ (1996) Angiotensin II type 2 receptor mediates programmed cell death. Proc Natl Acad Sci USA 93: 156–160 8552595 10.1073/pnas.93.1.156 PMC40197

[bib55] Yoshiji H, Yoshii J, Ikenaka Y, Noguchi R, Yanase K, Tsujinoue H, Imazu H, Fukui H (2002) Suppression of the renin–angiotensin system attenuates vascular endothelial growth factor-mediated tumor development and angiogenesis in murine hepatocellular carcinoma cells. Int J Oncol 20: 1227–1231 12012003

